# Spatio-temporal Prediction of the Malaria Transmission Risk in Minab District (Hormozgan Province, Southern Iran)

**DOI:** 10.1007/s11686-022-00598-2

**Published:** 2022-08-11

**Authors:** Abdolreza Salahi-Moghaddam, Habibollah Turki, Masoud Yeryan, Màrius V. Fuentes

**Affiliations:** 1 Infectious and Tropical Disease Research Center, Hormozgan Health Institute, Hormozgan University of Medical Sciences, Bandar Abbas, Hormozgan Iran; 2 Malaria Vector Unit, Minab Health Center, 17th Sharivar Ave, Minab, Hormozgan Iran; 3 Parasites and Health Research Group, Departament de Farmàcia i Tecnologia Farmacèutica i Parasitologia, Facultat de Farmàcia, Universitat de València, Av. Vicent Andrés Estellés s/n, 46100 Burjassot, València Spain

**Keywords:** Anopheline larvae, Environmental risk, GIS, Hormozgan province, Iran, Malaria

## Abstract

**Introduction:**

Malaria is the most important parasitic disease in tropical and subtropical regions, with more than 240 million cases reported annually. In Iran, indigenous cases occur in its south-eastern region. The aim of this study is to assess the environmental risk of malaria transmission in an endemic area of southern Iran.

**Methods:**

The study was carried out in Minab district (Hormozgan province, southern Iran), with the aim to assess the environmental risk of malaria, based on a spatio-temporal study, using Growing Degree Days (GDD)-based predictions, larval habitat ecology, MaxEnt spatial predictions and malaria transmission data.

**Results:**

The Gradient Model Risk index showed the highest malaria transmission risk period to be during January–April and October–December. The ecological conditions of water bodies of larval habitats of the four vector species (*Anopheles culicifacies*, *A. dthali*, *A. fluviatilis* and *A. stephensi*) were assessed, with *A. stephensi* being the most prevalent and the most widely distributed species.

**Conclusion:**

These findings, together with the MaxEnt *Anopheles* predictive distribution models, allowed identifying villages in danger of malaria transmission in Minab district. This spatio-temporal prediction of malaria transmission risk should be incorporated in the design of malaria control initiatives towards a local malaria early warning system. Moreover, the proposed transmission risk model can be extrapolated, at local scale, to other malaria endemic areas of tropical and subtropical regions.

## Introduction

According to WHO, 85 countries reported a total of 241 million malaria cases in 2020 [1], making malaria the most important parasitic disease in tropical and subtropical regions. Malaria is a severe parasitic disease in Iran, where transmission in the early twentieth century was facilitated by suitable conditions affecting between 4 and 5 million people of a total of 12 million people in that country and a total of 30–40% of all death were due to malaria [2]. Today, although a control programme to eliminate malaria by 2025 is being implemented in Iran, indigenous cases occur in the south-eastern part of the country [3].

In Iran, seven species of *Anopheles* Meigen, 1818 mosquitoes are malaria vectors, including *Anopheles culicifacies* s.l. Giles, 1901, *A. fluviatilis* s.l. James, 1902, *A. stephensi* Liston, 1901, *A. dthali* Patton, 1905, *A. sacharovi* (Favre, 1903), *A. maculipennis* (Meigen, 1818) and *A. superpictus* (Grassi, 1899) [4]. The spatial distribution of malaria and its main local vectors are restricted by climatic and geographical conditions determining the epidemiology of malaria at a local level [3–5]. Minab is an endemic malaria district of Hormozgan province, limited by the mountainous area of Bashagard to the north and by the Oman Sea to the south, turning this area into an unavoidable passage for crossing migrants coming from other malaria endemic areas. Therefore, there is a permanent presence of infected subjects in the area due to suitable climatic conditions for re-emerging malaria, even after successfully completing a temporary elimination scheme.

11,667 malaria cases (11,358 autochthonous and 309 imported) due to *Plasmodium vivax* (Grassi and Feletti, 1890) and/or *P. falciparum* (Welch, 1897) were reported in Iran between 2008 and 2010, although the number of malaria cases has decreased in recent years.

Minab health authorities are confident that malaria will be eradicated in the near future. Indigenous cases are becoming extremely rare, and they are mainly transmitted from imported cases influenced by some social factors [6]. However, predicting malaria in Minab district for the next years is rather difficult as transmission is affected, not only by drought and climate change, but also by agriculture and social-economic development with, for example, the possible construction of a new dam or another water supply project [3].

Larval conditions are increasingly recognized as having an influence on adult mosquito life history traits [7]. The existence of a strong association between the density and distribution of anopheline larvae and that of adults has been demonstrated. Some authors highlight the need to consider environmental variation of larval habitats to better understand transmission dynamics and control of vector-borne diseases, such as malaria [8]. Yet, control of larval mosquito populations is often convenient as the larvae are usually concentrated, relatively immobile, and occupy minimal habitat areas compared to adults able to rapidly disperse over large areas [9]. To apply larval source management activities efficiently, a comprehensive knowledge of the local ecology of anopheline larvae and their aquatic habitats is required [10].

On the other hand, the spatio-temporal distribution of anopheline larvae and thus, adult host-seeking and pathogen transmitting anophelines depend on parameters such as number, quality and size of potential larval habitats, their distance from blood meal sources and a wide range of other environmental factors [11]. Consequently, as comprehensive information on malaria epidemiology and vector bionomics will be necessary to eliminate malaria [10], an early warning system in this part of southern Iran, such as Geographic Information Systems (GIS), is meant to facilitate a tool in a preventive strategy. GIS projects, including the geo-referencing of larval habitats, outbreak locations, as well as the nearest road and villages or human settlements, should form part of a malaria elimination programme.

As the climatic conditions are key in the transmission of malaria in this endemic area, indices such as Growing Degree Days (GDD), which is based on suitable days for the growth of organisms in a month [12], are appropriate to estimate vector behaviour in a spatio-temporal context [13]. GDD is used on a national scale for malaria studies [14], but has not yet been applied in epidemiological studies in Iran. Moreover, recently, some ecological studies have demonstrated the usefulness of MaxEnt in estimating and predicting the spatial distribution of disease vectors, including snails, arthropods, even *Anopheles* species [15].

This study was conducted between 2014 and 2016 with the aim to assess the environmental risk of malaria in Minab district (Hormozgan province, South of Iran), based on a spatio-temporal study, analysing the relationship between malaria and climatic conditions, using GDD-based predictions, larval habitat ecology, MaxEnt spatial predictions and malaria transmission data.

## Materials and Methods

### Study Area

**Fig. 1:**
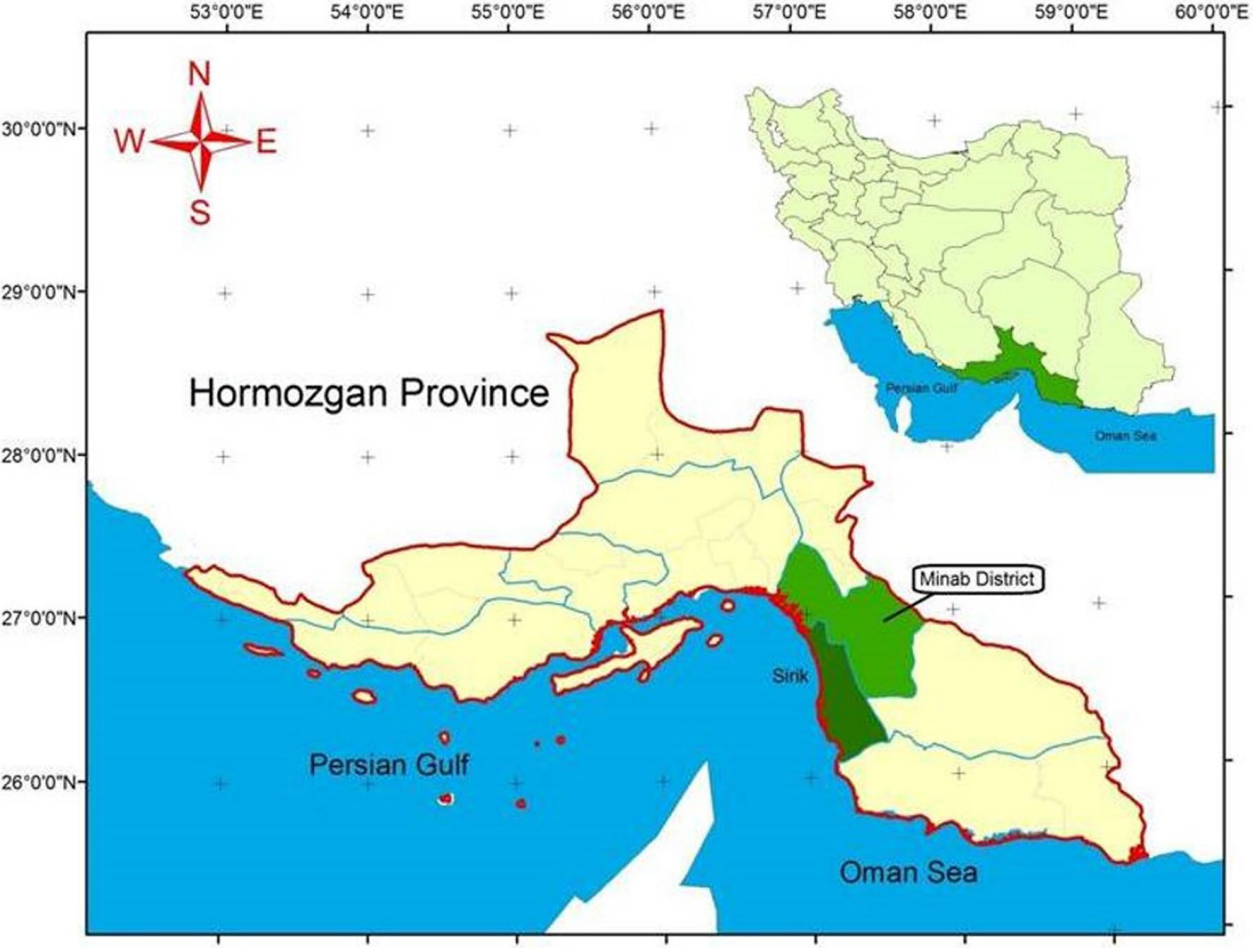
Minab and Sirik districts in the Hormozgan province of Iran

The study area is Minab district, in the province of Hormozgan (in the mid-south of Iran and in the northern part of the Strait of Hormuz), located between latitude 56.85 to 57.0 N and longitude 27.25 to 26.88 E (Fig. 1). This area covers 5393 km^2^, with a population of 705,234 inhabitants in 2016 according to its official website (http://www.minab.hormozgan.ir/). The average temperature in Minab is ≤ 20 ºC only during 3 months. However, the average temperature in the past 25 years was 27.4 ± 0.9 ºC, with the lowest temperature of 19.7 ± 1.2 ºC in February and the highest of 34.9 ± 0.7 ºC in July, respectively. Although the mean annual precipitation is only about 196.8 mm/year, this district is crossed by some rivers coming from the northern mountains.

A shapefile of Hormozgan province, including its rivers and water sources, was obtained from the Hormozgan governor. The Minab maps were generated from a master shapefile using ArcGIS 9.3, which was also used for all other GIS-based procedures.

### Climate Data

Three different types of climate data were used and/or checked for appropriateness: data generated since the 1960s from meteorological stations in the malaria endemic area, including Hormozgan, Kerman and Sistan-Baluchestan provinces, used to obtain a longitudinal perspective of the climate of the area (most of these studies were omitted); data of the Minab meteorological station between 1985 and 2010, used for ecological and epidemiological studies and for a better understanding of temporal conditions of malaria; and, in contrast, for the spatial part of the study, digital climate data obtained from WorldClim-Global Climate Data (http://www.worldclim.org/bioclim) were used.

Climatological variables of the meteorological station of Minab district from 1985 to 2010 were obtained from the Iranian Meteorological Research Centre (Iranian National Meteorology Organisation) (http://www.chaharmahalmet.ir/iranarchive.asp), including monthly and/or annual data of: mean, maximum and minimum temperatures (*T*) in ºC, relative humidity (RH), daily rainfall in mm, monthly maximum and mean wind speed in knots and monthly predominant wind.

Monthly potential evapotranspiration (PET) in mm was calculated by the equation

$$\mathrm{PET}=16\left(L/12\right)\left(N/30\right){\left(10T\alpha /I\right)}^{\propto }$$

where PET is mm/month; “*L*” is mean day length (in hours) of the month being calculated; “*N*” is number of days/month; “${T}_{a}$” is mean daily temperature (ºC), if this temperature is negative the value is substituted by 0; “α” and “*I*” are calculated from the equations below:

$$\propto =\left(6.75\times {10}^{-7}\right){I}^{3}-\left(7.71\times {10}^{-5}\right){I}^{2}+ \left(1.792\times {10}^{-2}\right)I+0.4923$$

$$I=\sum_{i=1}^{12}{\left({T}_{ai}/5\right)}^{1.514}$$

where ${T}_{ai}$ is 12 monthly mean temperatures [16].

A climadiagram based on the 1985–2010 climate data obtained from the Minab meteorological station was created for the study area [13, 17], delimiting the humid and dry period along the year. Moreover, the climadiagram also shows the optimum temperatures required for the sporogony cycle of *P. falciparum* (30 ºC) and *P. vivax* (25 ºC) [18], as well as the minimum and maximum temperatures for the optimum development of *Anopheles* mosquitoes (${T}_{\mathrm{min}}$–${T}_{\mathrm{max}}$), 20–30 ºC [3–5].

### Gradient Model Risk Index

The Gradient Model Risk (GMR) index, based on climatic conditions favouring the development of the parasite and vector, previously implemented to forecast the malaria transmission risk [12, 13, 19], was calculated using the following equation: *R*/PET

$$\mathrm{GDD}\times \frac{R}{\mathrm{PET}}, \mathrm{if}\, R/\mathrm{PET} \mathrm{is}>0.2 ;\mathrm{ if not}=\mathrm{GDD}$$

where GDD is growing degree days and *R* is rainfall. The threshold for surplus water (previously tested in Eritrea) was expected to be 0.2 (indicating that soil moisture was 20% saturated, the minimum soil moisture value consistent with malaria developmental cycles) [19], in the study area, being almost Afro-Tropical [20].

GDD is the degrees above a base value of 18 ºC and below an upper limit of 30 ºC. Below the lower limit, no development of *Plasmodium* spp., mainly *P. falciparum*, or *Anopheles* vectors occurs. GDD was calculated for each month (GDDm) by the equation

$$\mathrm{GDDm}=To\times N,\mathrm{ if}\frac{R}{\mathrm{PET}}>0.01; \mathrm{if not}\, \mathrm{GDDm}=0$$

where *N* is the number of days in the month and To is calculated by the equation

$${T}_{0}=T-{T}_{\mathrm{min}}, \mathrm{if} {T}_{\mathrm{min}}<T<{T}_{\mathrm{max}}; \mathrm{if not}\, {T}_{0}=0$$

where ${T}_{\mathrm{min}}$ and ${T}_{\mathrm{max}}$ are the minimum and the maximum temperature required for the development of *Plasmodium* Marchiafava and Celli, 1885 species, mainly *P. falciparum* (18–30 ºC), and *T* is the mean of temperature in that month.

The GMR index value established for one *Plasmodium* generation is 116 [19]. Consequently, values equal to or above 116 were considered for the onset of the malaria transmission risk.

### Malaria Data

Minab Health Centre Laboratory confirms, epidemiologically, records and treats all malaria cases in the district, as part of the malaria elimination programme in Iran, supervised by WHO. Autochthonous malaria cases, including those derived from imported cases, on record at Minab Health Centre since 2000 were considered as Minab malaria cases. Imported, recrudescent or possible transplacental cases were excluded. These data, originally recorded according to the Iranian calendar, were adapted to the Gregorian calendar.

### Larval Habitats

Important larval habitats including all permanent lagoons and pools with an area of more than approximately 10 m^2^ were identified in Minab district and in some neighbouring sites of Sirik district. Their geographical coordinates were recorded using a GPS and located on a local map. Parameters of the water sources, such as pH, Total Dissolved Salts (TDS) in ppm-mg/l, temperature in ºC, and dissolved oxygen in ppm, were registered using a portable multimeter. Temporary larval habitats were not considered.

Larval density was calculated in the field for each sampled larval habitat, counting collected larvae with up to ten dips with a 250-ml volume ladle. Water samples were taken at each larval habitat and transported to the entomological laboratory of Minab Health Centre where anopheline larvae were specifically identified.

### MaxEnt Modelling

WorldClim-Global Climate Data (http://www.worldclim.org/bioclim) provides free climate data often used for ecological modelling, mainly in species distribution modelling. Bioclimatic variables, derived from the monthly temperature and rainfall data from meteorological stations over a 50-year period, from 1950 to 2000, generate meaningful variables, representing annual trends, seasonality and extreme or limiting environmental factors. Images corresponding to 19 bioclimatic variables (Table 1) and altitude, with a spatial resolution of about 1 km^2^, were downloaded from the WordClim website.

**Table 1:** Environmental and climatic variables used in the MaxEnt modelling to predict the occurrence of *Anopheles* spp. larvae in Minab district

BIO1 = Annual mean temperature
BIO2 = Mean diurnal range (Mean of monthly (max temp - min temp))
BIO3 = Isothermality (BIO2/BIO7) (* 100)
BIO4 = Temperature seasonality (standard deviation *100)
BIO5 = Max temperature of warmest month
BIO6 = Min temperature of coldest month
BIO7 = Temperature annual range (BIO5-BIO6)
BIO8 = Mean temperature of wettest quarter
BIO9 = Mean temperature of driest quarter
BIO10 = Mean temperature of warmest quarter
BIO11 = Mean temperature of coldest quarter
BIO12 = Annual precipitation
BIO13 = Precipitation of wettest month
BIO14 = Precipitation of driest month
BIO15 = Precipitation seasonality (coefficient of variation)
BIO16 = Precipitation of wettest quarter
BIO17 = Precipitation of driest quarter
BIO18 = Precipitation of warmest quarter
BIO19 = Precipitation of coldest quarter
Altitud

Maximum entropy (MaxEnt) modelling was used to predict the distribution of the four dominant *Anopheles* species in the study area [21–23], based on the finding of their most appropriate ecological niches, from the analysis of the 19 bioclimatic variables and altitude and the known presence of *Anopheles* spp. in water bodies of Minab district. MaxEnt 3.3 software, available from https://www.cs.princeton.edu/~schapire/maxent/maxent-submit.cgi, was used to generate maps showing the predicted distribution of *Anopheles* spp., malaria vectors, in the study area, plotted with a spatial resolution of 1 km.

The accuracy of the predictive distribution models was measured using the area under the curve (AUC) with values ranging from zero to one: values below 0.5 indicate low model predictive ability, while values between 0.5 and 1 indicate high predictive performance of the model. It estimates the probability of a test pixel to be correctly predicted as suitable for species presence as opposed to the probability of a randomly selected pixel of the map [24]. The influence of the 20 environmental and climatic variables on the larval habitat distribution was demonstrated by: the analysis of variable contributions, which shows the environmental variables used in the model, their percent contribution and their permutation importance; and the Jackknife of regularized training gain (Jackknife test), which shows the training gain of each variable if the model was run in isolation, and compares it to the training gain with all the variables.

Four models were constructed: *A. culicifacies*, *A. fluviatilis*, *A. dthali* and *A. stephensi*.

To combine MaxEnt and the ArcGIS map, the ASCII file of the MaxEnt output file was transformed to raster layer, using the “Ascii to Raster” toolbox and its values were extracted using “extract to point”. Band 3 (blue) was used as it is density of high risk, while band 1 (red) is density of low risk and band 2 (green) is intermediate condition. We used band 3 which is vast from 0 to 255, being the opposite of band 1. Endangered villages, close to malaria transmission locations, i.e., less than 2 kms distance from the centre of the epidemic area, received this value, thus generating a map of malaria transmission and vector distribution, considering the larval ecology. This map was used for the spatial prediction of malaria transmission.

### Statistical Analysis

In addition to the statistical analysis carried out by the MaxEnt software, the Rho Spearman correlation was used to assess the potential relation between monthly malaria cases and GMR values, as well as between the number of *Anopheles* larvae collected belonging to each of the four species and the ecological parameters of the water bodies. Moreover, Binary logistic regression was used to demonstrate the potential association (presence/absence) between the larval species in the water bodies analyzed. Statistical significance was established at *P* < 0.05.

## Results

### Climate Conditions

**Fig. 2:**
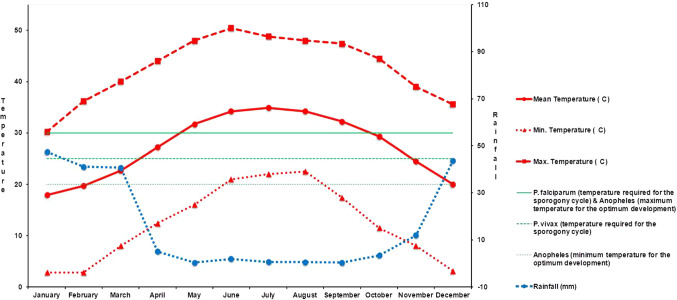
Climadiagram of Minab district showing: minimum, maximum and mean temperatures; rainfall; optimum temperatures required for the sporogony cycle of *Plasmodium falciparum* (30 ºC) and *P. vivax* (25 ºC); and the minimum and maximum temperatures for the optimum development of *Anopheles* mosquitoes (20 ºC–30 ºC)

The climadiagram of the study area (Fig. 2) shows a short wet season from December to March and a dry season for the rest of the year. Moreover, the optimum temperatures required for the sporogony cycle of *P. falciparum* (30ºC) and *P. vivax* (25 ºC) are within the monthly values of minimum and maximum temperatures along the entire year, being below the mean temperature values during the period from May to October. The February–May as well as the October–December periods register the optimum temperatures for the development of *Anopheles* mosquitoes (20–30 ºC).

### Gradient Model Risk Index

**Fig. 3:**
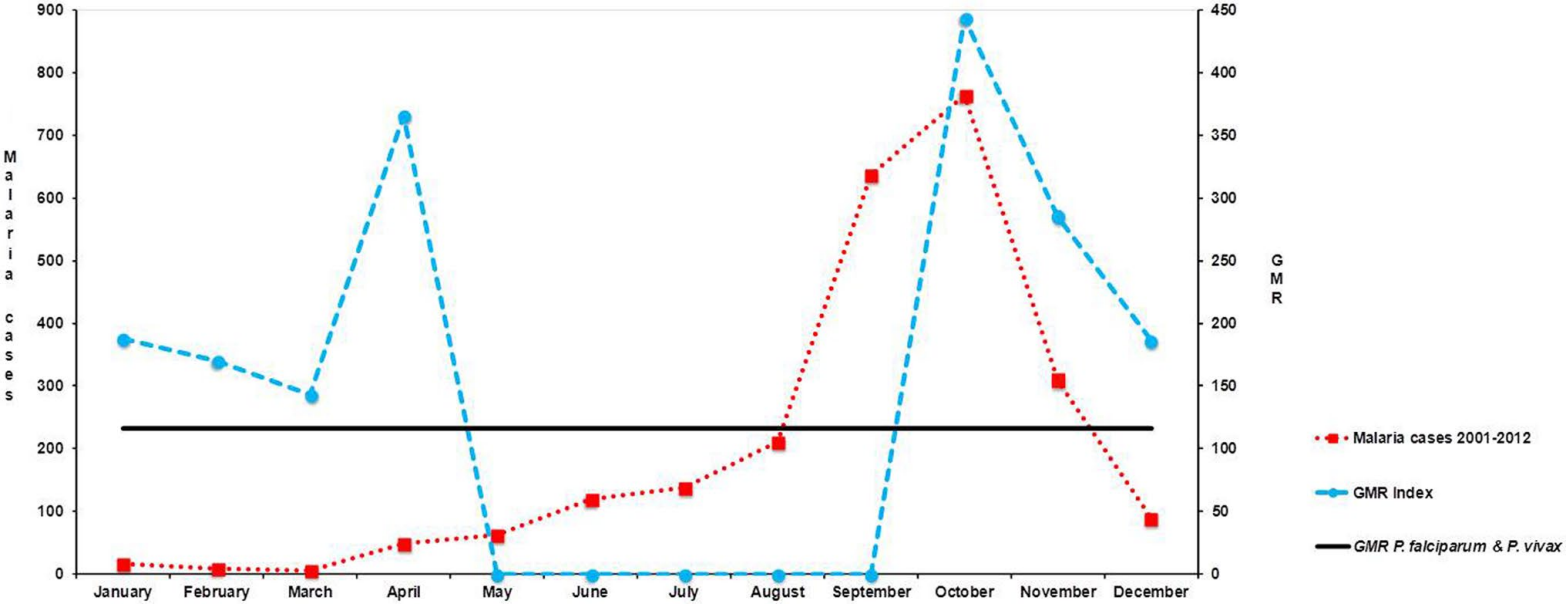
Accumulated monthly malaria cases from 2001 to 2012 and Gradient Model Risk (GMR) in Minab district, with the interpolation of the minimal GMR required for *Plasmodium falciparum* and *P. vivax* transmission (116)

GMR index values for the threshold of 0.2 (Fig. 3) show the likelihood of the onset of the malaria transmission risk, allowing the development of several *P. falciparum* and/or *P. vivax* generations during the January–April and October–December periods.

### Malaria Cases

**Fig. 4:**
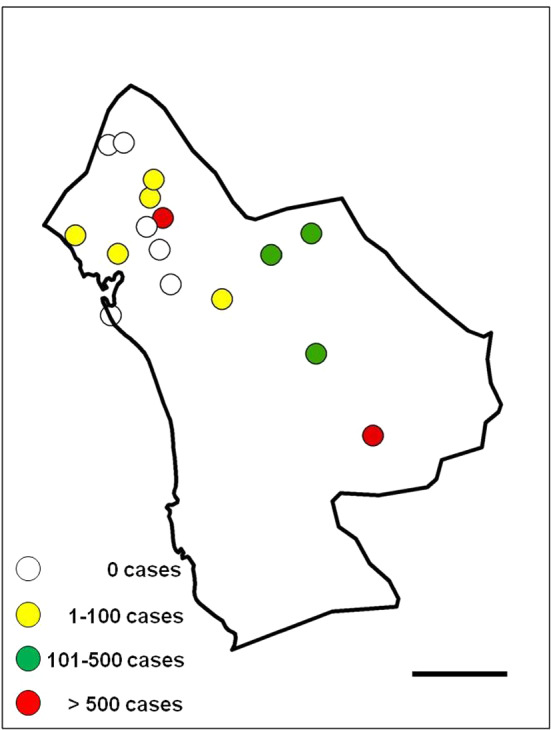
Map of Minab district representing the distribution of malaria cases between 2001 and 2012 reported by health centres. Scale bar = 25 km

During the 2001–2012 period, 2864 indigenous malaria cases were reported in Minab district, 2820 (98.5%) due to *P. vivax* and 44 (1.5%) due to *P. falciparum*. Considering transmitted cases, indigenous malaria outbreaks may reach their peak at the end of summer/early autumn. Although there are malaria cases along the entire year, the main outbreak period of malaria transmission with more than 200 cases is the quarter September–November (Fig. 3). The spatial distribution of malaria transmission in Minab district, including indigenous cases as well as those transmitted from imported cases, is shown in Fig. 4.

Spearman’s Rho between malaria cases and GMR index values did not show any significant correlation. However, if the five monthly values in which GMR = 0 were omitted (period May–September), Spearman’s Rho showed a positive statistically significant correlation between the index and malaria cases (Rho = 0.82; *P* = 0.02).

### Ecology of Anopheles Larvae

Important larval habitats including all permanent lagoons and pools with an area of more than approximately 10 m^2^ were identified in Minab district and in some neighbouring sites of Sirik district.

**Fig. 5:**
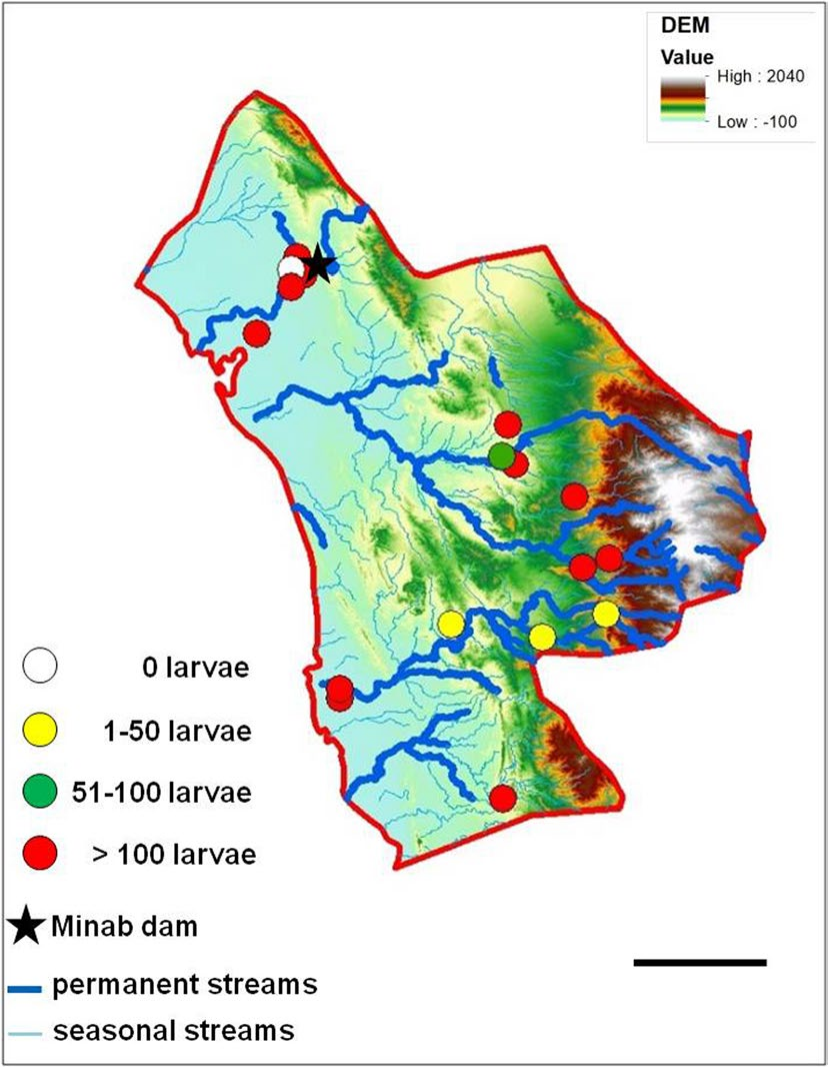
Altitudinal map (by Digital Elevation Model) and streams map of Minab district representing the distribution of larval habitats and collected larvae. Scale bar = 25 km

**Fig. 6:**
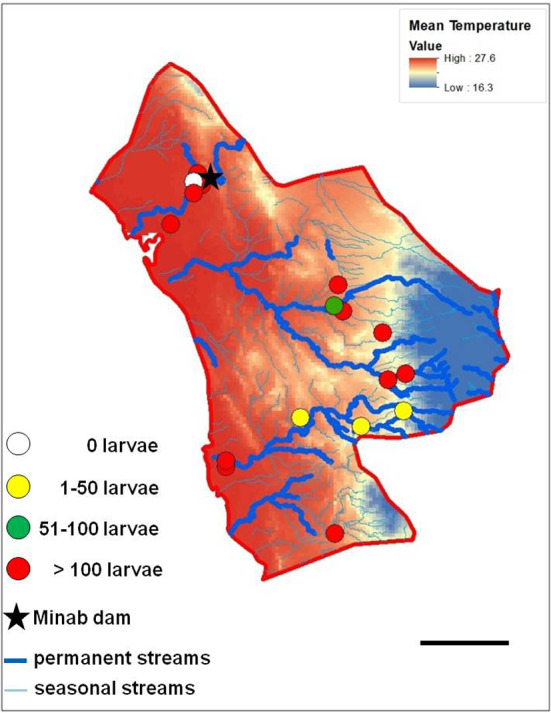
Annual mean temperature and streams map of Minab district representing the distribution of larval habitats and collected larvae. Scale bar = 25 km

After identifying and checking permanent water bodies for anopheline larve in the study area, 18 larval habitats were selected and their ecological conditions were assessed. Most larval habitats can be divided in two spatial groups: larval habitats near the “Bashagard elevations” in the east and larval habitats in the west around the river Minab and the Minab dam (Figs. 5, 6). Malaria vector species considered in the study were: *A. culicifacies*, *A. dthali*, *A. fluviatilis* and *A. stephensi*.

**Fig. 7:**
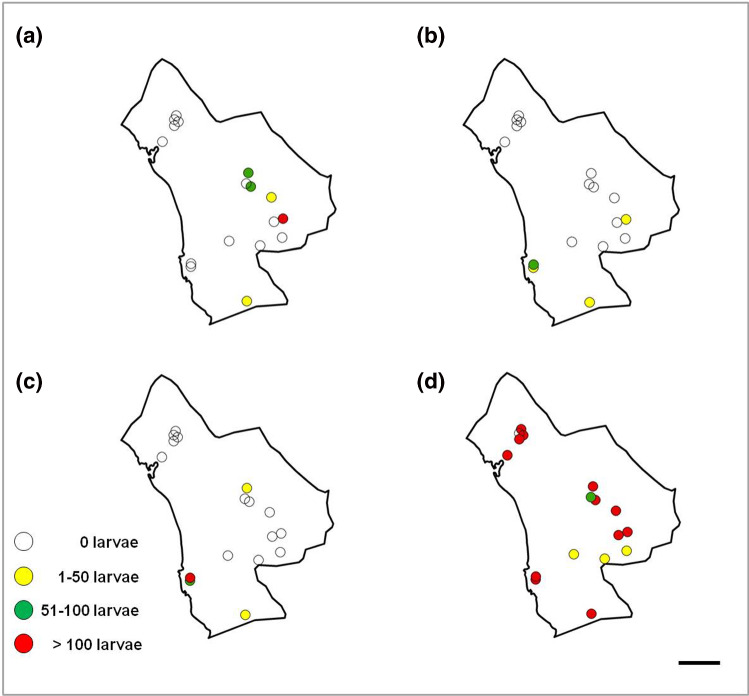
Distribution of *Anopheles* larval habitats and collected larvae in Minab district: **a** *A. culicifacies*; **b** *A. dthali*; **c** *A. fluviatilis*; **d** *A. stephensi*. Scale bar = 25 km

The anopheline with the most prevalent and widest distribution of larvae in the study area was *A. stephensi*, present in 17 of the 18 selected water bodies. *Anopheles culicifacies*, more prevalent in the eastern part, close to the mountainous area of Bashagard, was present in five water bodies. *Anopheles dthali* and *A. fluviatilis*, present in four water bodies and sharing three of them (*χ*^2^ = 7.37; *P* = 0.01), were more prevalent in the flatter area close to the coast of the Oman Sea (Fig. 7).

The analysis of the parameters of the water bodies where larvae were collected (Table 2) shed light on the preferences of each mosquito species. *Anopheles culicifacies* developed better at a temperature between 24 and 30 ºC, with a preference of a pH value ≥ 8.35 and a TDS value ≥ 550 (the quantity of larvae correlated positively with the TDS values; Rho = 0.51; *P* = 0.04). *Anopheles dthali* and *A. fluviatilis* developed better at a temperature between 24 and 28 ºC, with a preference of a pH value ≥ 8.3 and a TDS value of ≤ 410 and ≤ 370, respectively. *Anopheles stephensi* developed better at a temperature between 28 and 34 ºC, with its population plummeting at temperatures below 28 ºC, with a preference of a pH value ≥ 8.0 (Rho = 0.58; *P* = 0.02) and a TDS value ≥ 420. Concerning the dissolved oxygen parameter, two findings are noteworthy: only *A. culicifacies* larvae were not found in water bodies with a high oxygen concentration; and the quantity of larvae correlated positively with dissolved oxygen values in *A. dthali* and *A. fluviatilis*, but only being statistically significant (Rho = 0.61; *P* = 0.01) in the former.

**Table 2:** Ecological variables in *Anopheles* spp. larval habitats. n = number of water bodies in which the larvae were present; TDS (total dissolved salts)

*Anopheles* spp.	*n*	pH	TDS (ppm-mg/l)	Oxygen (ppm)	Temperature (ºC)
*A. culicifacies*	5	8.35–8.65	3.83–6.86	0.70–1.12	24.3–34.4
*A. dthali*	4	8.10–8.65	1.70–3.83	0.89–98.80	22.6–34.4
*A. fluviatilis*	4	8.10–8.56	1.70–5.58	0.70–98.80	22.6–34.4
*A. stephensi*	17	7.80–8.65	1.70–18.37	0.37–98.80	22.6–37.0

### MaxEnt Predictive Distribution Models

**Fig. 8:**
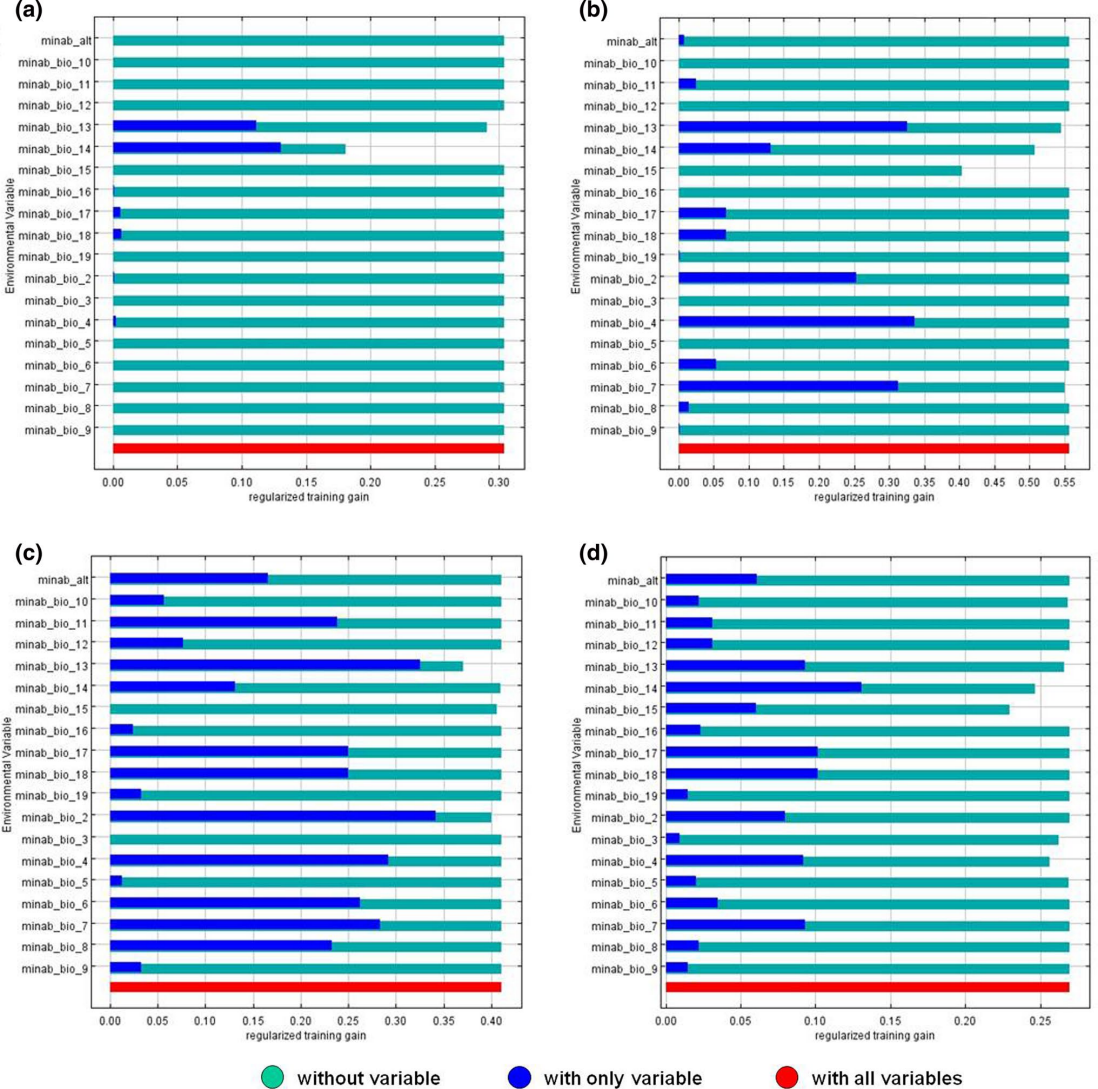
Jackknife test of variable importance contribution to the MaxEnt model to determine *Anopheles* larval habitats in Minab district: **a** *A. culicifacies*; **b** *A. dthali*; **c** *A. fluviatilis*; **d** *A. stephensi*

MaxEnt predictive distribution models were generated for each of the four *Anopheles* larvae species analysed. Statistical results concerning the influence of environmental and climatic variables on the predictive larval habitats, i.e., percent variable contribution and their permutation importance, as well as the Jakknife test, are shown in Table 3 and Fig. 8, respectively.

**Table 3:** Analysis of the variable contributions to the MaxEnt model to determine *Anopheles* larval habitats in Minab district

Variable	*Anopheles *especies	Percent contribution	Permutation importance
BIO3 = Isothermality	*A. stephensi*	1.4	8
BIO4 = Temperature seasonality	*A. fluviatilis*	42.2	0
	*A. dthali*	52.9	0
	*A. stephensi*	15.1	11.6
BIO5 = Max temperature of warmest month	*A. stephensi*	3.1	3.8
BIO6 = Min temperature of coldest month	*A. fluviatilis*	6	0
BIO7 = Temperature annual range	*A. dthali*	0.9	39.5
BIO8 = Mean temperature of wettest quarter	*A. fluviatilis*	8	0
	*A. stephensi*	1	0.2
BIO-9; Mean temperature of driest quarter	*A. culiciphacies*	14	15.8
	*A. stephensi*	0.4	2.4
BIO10 = Mean temperature of warmest quarter	*A. stephensi*	0.7	5.6
BIO11 = Mean temperature of coldest quarter	*A. stephensi*	3.2	4.7
BIO13 = Precipitation of wettest month	*A. culiciphacies*	1.4	5.3
	*A. fluviatilis*	14.4	21.7
	*A. dthali*	1.3	1.8
	*A. stephensi*	0.6	11.2
BIO14 = Precipitation of driest month	*A. culiciphacies*	52.4	31.2
	*A. fluviatilis*	21	27.1
	*A. dthali*	24.3	25.5
	*A. stephensi*	49.9	25.5
BIO15 = Precipitation seasonality (coefficient of variation)	*A. fluviatilis*	1.1	2.8
	*A. dthali*	20.5	33.2
	*A. stephensi*	17.5	6.9
BIO17 = Precipitation of driest quarter	*A. fluviatilis*	0.6	0
BIO18 = Precipitation of warmest quarter	*A. fluviatilis*	2	23.1
BIO19 = Precipitation of coldest quarter	*A. culiciphacies*	32.2	47.7
	*A. stephensi*	7.1	20.2
Altitude	*A. fluviatilis*	3.4	0

**Fig. 9:**
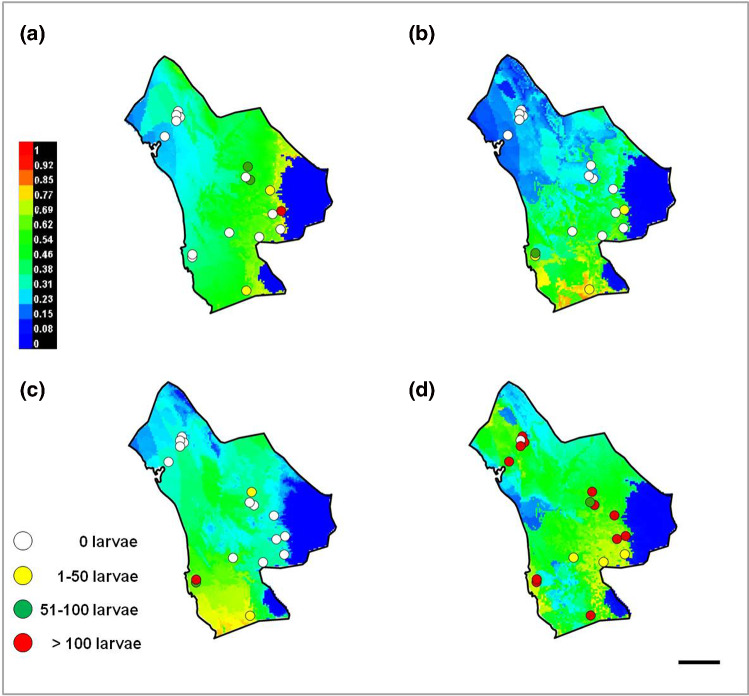
MaxEnt model predictive map of *Anopheles* larval habitats overlapped with the distribution of known *Anopheles* larval habitats and collected larvae in Minab district: **a** *A. culicifacies*; **b** *A. dthali*; **c** *A. fluviatilis*; **d** *A. stephensi*. Scale bar = 25 km

The AUC of the predictive model was 0.83 for *A. culicifacies*, 0.94 for *A. dthali*, 0.84 for *A. fluviatilis* and 0.86 for *A. stefensi*. Although other variables showed an important influence, the most influential variable on *A. dthali* and *A. fluviatilis* was Bio4 (temperature seasonality), while the most important influence exercised on *A. culicifacies* and *A. stephensi* was Bio 14 (precipitation of driest month). Moreover, a map was also generated for each *Anopheles* species, showing the predicted larval habitat distribution (Fig. 9).

**Fig. 10:**
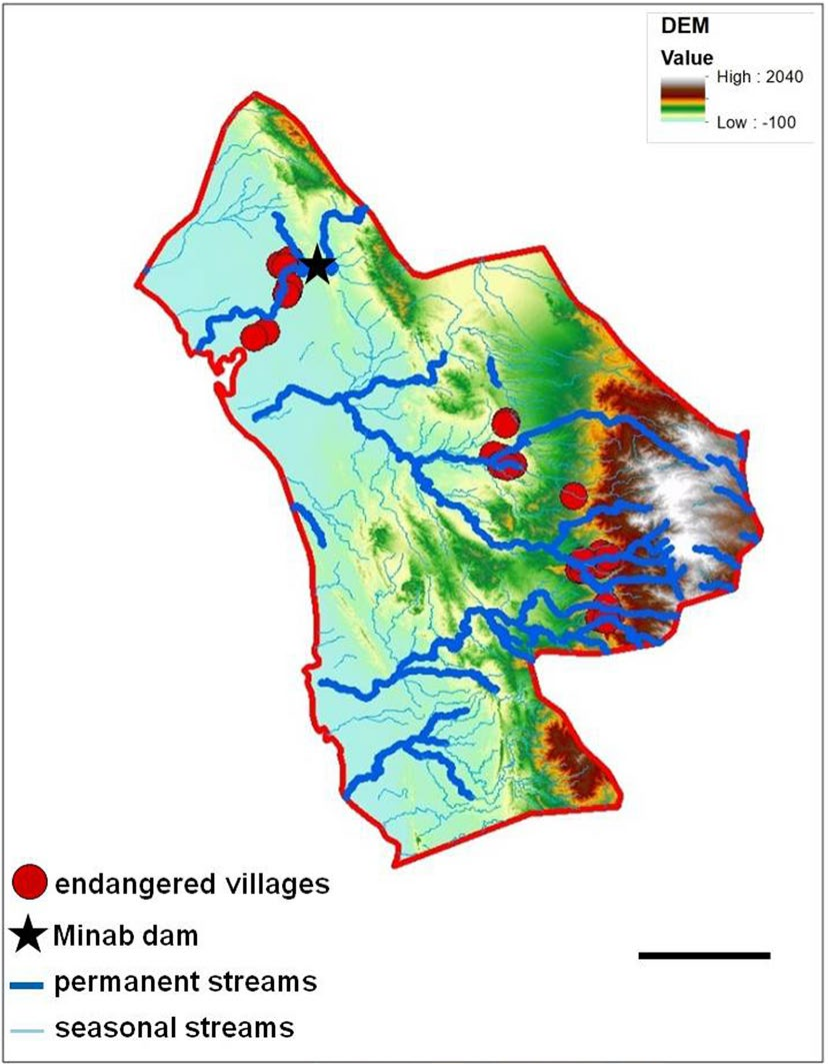
Endangered villages for malaria transmission in Minab district with permanent and seasonal streams in the background. Scale bar = 25 km

A combination map of important larval habitats and places with ecological potential for the existence of vectors allowed identifying villages in danger of malaria transmission (Fig. 10), using, for more preventive action, a flight distance buffer of 2 kms for the *Anopheles* spp.

## Discussion

According to previous local epidemiological malaria studies [25], August–November comprises the most important malaria transmission period, coinciding with the present study carried out in Minab district, in which the most important transmission period was found to be in September and October.

Although other studies have also reported the scarce presence of *A. superpictus* in the mountainous areas of Minab and the neighbouring district of Bashagard [22, 26], the epidemiology of malaria in Minab district is based on the presence of four *Anopheles* species, *A. stephensi*, *A. culicifacies*, *A. dthali* and *A. fluviatilis*, which agrees with the present results and previous studies carried out in this district. Our findings are in agreement with those of Soleimani in Minab and Azizi on the ecology of larval habitats in Bandar Abbas and Bashagard districts as well as other ecological research concerning *Anopheles* spp. and specifically *A. stephensi* [27–29].

Malaria vectors, especially the main local vector *A. stephensi*, develop almost during the entire year, showing two annual peaks of activity in May and October, but with a largely diminished activity from June to September [30]. According to the monthly values of the GMR index, parasite development along time diminishes in May, just 1 month before the vector population falls, and is reactivated in October, just 1 month after the recovery of vector activity. Moreover, malaria transmission rises dramatically in September and the outbreak period begins. The GMR index values also agree with the ecology of *A. dthali* in Bashagard [31], where the peaks of *Anopeheles* activity are registered in April and October [25], while the most important malaria transmission period occurs in September and October [32]. Remarkably, main malaria outbreaks begin in September, coinciding with the increasing GMR index values as well as the optimum conditions for *Anopheles* development. This fact is supported by the positive correlation between GMR index values and malaria cases. Our temporal prediction of malaria transmission is based on the first GDD and GMR index estimation applied in Iran. As Minab district is located in the tropical area of Iran [20], the use of the optimal temperature for sporogony, 25 ºC for *P. vivax* and 30 ºC for *P. falciparum* [18], may seem more suitable for prediction. This may explain the few malaria cases reported during the period from January to March in contrast to the GMR index prediction for these months. The GMR index takes into account a base value of 18 ºC not of 25 ºC. On the other hand, in the period between May and September, the GMR index values are = 0 because the mean temperature is > 30 ºC and rainfall is very scarce, without any surplus water. These conditions, at least theoretically, do not allow malaria transmission due to the absence of the optimal conditions for both, the development of *Anopheles* and the sporogony cycle. However, a trickle of malaria cases can occur due to various factors: some infected mosquito females remaining, for example, females of the main malaria vector *A. setphensi*, considered endophagous and endophilic; the existence of optimal conditions in some areas of the district where transmission can occur below the mean temperature; the report of cases related to immigrants, etc. As these potential causes of a scarce but actual malaria transmission cannot be predicted by the GMR index, the period with values = 0 should be omitted from the Rho test.

Malaria in the south of Iran has two peaks, in April and October, but with the efforts of the malaria elimination programme, although peaks of mosquitoes are seen in the predicted months, the density of vectors is too low for malaria outbreaks in spring. However, the vector population in summer is higher than in winter, causing malaria outbreaks in the late summer and autumn. Moreover, the period from January to March presents GMR index values lower than in the malaria outbreak period (October–December), and registered mean temperature values lower or very close to the minimum temperature for the development of mosquito vectors and parasites, corresponding to the season with fewer malaria cases.

Water collection habitats where mosquito larvae development takes place have been characterized for some *Anopheles* species in Iran, including the most recent studies carried out in several districts of Hormozgan province [29, 30, 33]. However, the present study includes additional parameters such as TDS, oxygen, pH and temperature, making the comparison of our results with other similar ones difficult.

The alkalinity found in larval habitats in Minab district is compatible with the pH values reported in the neighbouring districts of Bandar Abbas, Bashagard and Rudan [26, 28, 34]. *Anopheles stephensi* is not only the most prevalent and most important malaria vector in south-eastern Iran, but also in Minab and Jask districts [23, 29]. Its larvae, also according to previous studies [3, 4, 33], are resistant to ecological changes of the environment and may breed in a wide variety of ecological habitats, with the widest range of temperature, salinity, pH and dissolved oxygen in the water bodies. The wide range of water salinity found in the breeding habitats of *A. stephensi* was previously reported by Vatandoost et al. [30] who found these larvae in water habitats which sometimes reached or even exceeded that of seawater. However, the water parameters required for larval development of the three other *Anopheles* species are relatively similar, with the exception of the oxygen concentration which has a very narrow range in the habitats of *A. culicifacies*, and similar to those reported in other neighbouring districts [26, 31, 34].

Our findings on the ecology of *Anopheles*, mainly those on *A. stephensi* and *A. culicifacies*, agree with the malaria risk map published by Ahmadian-Merj et al. [35] using satellite images. Figure 7d shows the distribution of *A. stephensi* mainly in two zones: one in the mountainous area and another in the plain (low altitude area), prompting the question about the suitable ecological niche of *A. stephensi*: the mountains or the plane? Considering the results of Oshaghi et al. (2006) [27], these two *A. stephensi* populations belong to two mosquito types: *A. stephensi mysorensis* Sweet and Rao, 1937 in the mountainous area, adjacent to Bashagard for the eastern population, and intermediate forms for the northern population. Moreover, and according to previous studies [3], the predictive distribution model shows that *A. culicifacies* has more affinity for the eastern part. In this area, precipitation of driest month (BIO14) and precipitation of coldest quarter (BIO19), two of the most influent variables, offer the best conditions for this mosquito species.

Our results strongly agree with other findings on malaria conditions in the western district of Bandar Abbas, where the most important collected vectors were *A. stephensi*, *A. fluviatilis* and *A. dthali* [6]. However, these authors did not report the presence of *A. culicifacies*, possibly because Bandar Abbas is located more to the west. Nevertheless, the ecology of *A. culicifacies* in Minab district coincides with that reported in other provinces of southern Iran and Bashagard and Jask districts [25, 36]. Moreover, the bio-ecology of malaria vectors in Minab also agrees with that reported in the south-eastern district of Jask [23], where nearly 8400 larvae were collected, although that study focused on population dynamics and insecticide resistance of *Anopheles* species rather than on spatio-temporal analysis of malaria in that endemic area. Finally, our results present other differences and similarities with respect to the studies carried out in some close districts: in Bandar Abbas, malaria outbreaks reach their peak in August [6], just when malaria fluctuation starts to rise in Minab; the prediction made from GDD agrees with findings in Jask district [23], where the peak of the dominant vector, *A. stephensi*, was also in April and October.

The accuracy of the model has been evaluated with regard to three different perspectives: temporal, spatial and the characterization of water bodies for larval development. The correlation between the GMR index and malaria cases supports the temporal prediction of outbreaks; MaxEnt model results support the spatial distribution of the four *Anopheles* species larvae, predicting their most appropriate ecological niches; and the correlations between physicochemical parameters show the water body preferences for each mosquito species. Moreover, villages in danger of malaria transmission have been identified.

The model agrees with actual malaria outbreaks. However, once the impact of the national malaria elimination programme affects vector ecology, and in the context of a changing scenario due to global warming and/or climate change, this model will be re-evaluated and adjusted accordingly. Until then, a follow-up study to re-confirm its validity will be recommended.

## Conclusion

The first local malaria risk map of Minab district has been designed considering meteorological data and GMR-based predictions, mosquito epidemiological data as well as larval habitat ecology, malaria transmission data, and MaxEnt spatial predictions. This spatio-temporal prediction of malaria transmission risk should be incorporated in the design of malaria control initiatives by Minab health authorities towards a local malaria early warning system. Moreover, the proposed transmission risk model can be extrapolated, on a local scale, to other malaria endemic areas of tropical and subtropical regions.

## Data Availability

Not applicable.
